# The UspIC: Performing Scan Matching Localization Using an Imaging Sonar

**DOI:** 10.3390/s120607855

**Published:** 2012-06-08

**Authors:** Antoni Burguera, Yolanda Gonzàlez, Gabriel Oliver

**Affiliations:** Departament de Matemàtiques i Informàtica, Universitat de les Illes Balears, Ctra. de Valldemossa Km. 7.5, 07122 Palma de Mallorca, Illes Balears, Spain; E-Mails: y.gonzalez@uib.es (Y.G.); goliver@uib.es (G.O.)

**Keywords:** imaging sonar, underwater robotics, localization, scan matching

## Abstract

This paper presents a novel approach to localize an underwater mobile robot based on scan matching using a *Mechanically Scanned Imaging Sonar* (MSIS). When used to perform scan matching, this sensor presents some problems such as significant uncertainty in the measurements or large scan times, which lead to a motion induced distortion. This paper presents the *uspIC*, which deals with these problems by adopting a probabilistic scan matching strategy and by defining a method to strongly alleviate the motion induced distortion. Experimental results evaluating our approach and comparing it to previously existing methods are provided.

## 1. Introduction

Thanks to recent technological advances, the sub-aquatic world is more accessible for exploration, scientific research and industrial activity. When the tasks involved in missions are repetitive, hazardous or too long to be carried out by divers or human guided vehicles, the use of unmanned vehicles becomes more suitable.

At present, *Remotely Operated Vehicles* (ROVs) are commonly used in a variety of applications such as surveying, scientific sampling, rescue operations or infrastructure inspection and maintenance. Trying to overcome some of the intrinsic limitations of ROVs, such as their limited operative range or the need of a support vessel, *Autonomous Underwater Vehicles* (AUVs) are progressively being introduced.

### 1.1. Underwater Sensing

Improving the sensorial capabilities of underwater vehicles is a key point to increase the variety and the feasibility of missions that can be carried out by ROVs and AUVs. Optical sensors, such as laser range finders or cameras, can provide dense information updated at high speed and they are commonly used in many terrestrial and aerial robotic applications [1-4]. However, due to the interaction between water and electromagnetic waves, optical systems are very problematic in the underwater domain. Light attenuation and scattering, non-uniform lighting and shadows, colour filtering or suspended particles are frequent difficulties when using optical sensors underwater [5].

To the contrary, acoustic sensors have interesting properties in these scenarios. For example, sound propagates faster in water than in air, and it is able to travel larger distances than light in this media. Because of that, acoustic sensors such as sonars are the most common choice for AUVs [6-9].

There are several types of sonars that can be used in underwater robotics [10], ranging from simple and inexpensive *echo sounders* that provide single range measurements, to complex and expensive *multi-beam imaging sonars* and *side-scan sonars*, which provide echo intensity or range profiles at high frequencies.

Somewhere in between lies the *Mechanically Scanned Imaging Sonar* (MSIS). This device has a transducer which emits fan-shaped beams, being able to perform, in most configurations, 360° scans by means of a mechanical rotation. They provide echo intensity profiles and can be placed to scan the AUV horizontal plane. The mechanical rotation leads to a large scan time, which is said to be one of the most important drawbacks of these sensors, especially when they are mounted on AUVs.

In spite of these drawbacks, MSIS sensors have interesting properties when used in AUVs. On the one hand, these sensors are cheaper than multi-beam or side-scan devices. On the other hand, they can be easily mounted on the vehicle in different configurations. For example, they can be easily used to scan the horizontal or the vertical AUV plane.

### 1.2. Underwater Scan Matching

Nearly all underwater robotic tasks require some knowledge of the robot location in the environment. For example, in power or communications cable inspection [11], the robot has to estimate its own pose in order to track the cable and properly keep records of damaged areas. Also in sampling tasks [12], either biological, archaeological or geological, the knowledge of the robot pose is required to properly map the sampling area. Of course, survey and mapping missions also require accurate robot pose information. The problem of estimating the robot pose is known as *localization*.

The literature approaches the problem of localization from different perspectives, ranging from simple dead reckoning to methods relying on complex representations of the environment. As a matter of fact, the simultaneous estimation of the robot pose and the environment map has been one of the most active research areas in the past years and is still the focus of several studies. It is the so-called *Simultaneous Localization and Mapping* (SLAM) [13,14]. Although a wide variety of sensors has been used to perform localization, most of the research has been traditionally conducted using accurate laser scanners. Recent trends focus on the use of cameras, both monocular and stereo, as well as other novel optical sensors.

As stated previously, optical sensors are not a good choice in underwater robotics and acoustic sensors are preferred. Unfortunately only a few studies on localization deal with these sensors in the underwater domain. Additionally, underwater environments pose several difficulties to localization. For example, GPS signal is not available. Also, most underwater environments are unstructured, making it unfeasible to use conventional feature-based or line-based approaches.

Scan matching approaches to localization are based on matching range scans neither making any assumption about their shape nor searching features [15]. In other words, scan matching approaches rely on the raw data provided by range sensors. First attempts to perform mobile robot localization by matching successive range scans were inspired by the computer vision community. A standard approach to image registration is the *Iterative Closest Point* (ICP). Although the name ICP was firstly introduced by Besl and McKay [16], very similar ideas were presented by other authors such as Chen and Medioni [17] or Zhang [18].

The ICP concepts were introduced in the mobile robot localization context by Lu and Milios [19]. These authors proposed some changes to the original algorithm to make it more suitable for robotic applications. As a matter of fact, due to the great success of this approach, many other scan matching algorithms rely on the same basic structure, defining the *ICP-based* family of algorithms. Algorithms such as the *Iterative Dual Correspondence* (IDC), also proposed by Lu and Milios, the *Metric-Based ICP* (MbICP) [20], the *probabilistic Iterative Correspondence* (pIC) [21], the *Polar Scan Matching* (PSM) [3], *the Point to Line ICP* (PLICP) [22] or the proposal by Pfister *et al.* [23] constitute well known examples of ICP-based algorithms.

Additionally, different researchers have proposed alternative approaches to scan matching not relying on the ICP concepts. The *Normal Distributions Transform* (NDT) [24], the probabilistic scan registration [25], the scan correlation [26] and the *Likelihood Field with Sum of Gaussians* (LF/SoG) [27] are good examples of these alternatives to ICP-based.

In general, scan matching algorithms require dense sets of accurate range readings to obtain reliable motion estimates. That is why laser sensors are often used in the context of terrestrial scan matching. Besides, when sonar sensors are used in the context of scan matching, some difficulties arise. In particular, performing scan matching with an MSIS poses two important problems. Firstly, this sensor does not provide range measurements but echo intensity profiles. Accordingly, the sensor information has to be processed before being used in the scan matching context [28]. Secondly, the scan time of an MSIS is not negligible. Because of this, it can not be assumed that the robot remains static while the scan is being obtained.

A few studies exist dealing with these problems. For example, [29,30] propose partial solutions. The former takes into account the MSIS characteristics, but it performs feature based SLAM instead of scan matching. The latter performs sonar scan matching and deals with problems similar to the aforementioned ones, but using an array of terrestrial Polaroid sonar instead of an underwater MSIS. Moreover, some recent studies [31–33] show the feasibility of underwater scan matching using an MSIS.

Our goal is to provide self-localization capabilities to an AUV. To this end, this paper proposes a framework to perform scan matching in underwater environments using an MSIS. This framework includes processes to deal with the aforementioned problems of MSIS sensing. Also, a probabilistic scan matching method previously tested on terrestrial sonars, the *sonar probabilistic Iterative Correspondence* (spIC) introduced in [30], is used to perform the matching. Because of this, the framework introduced in this paper will be referred to as the *underwater spIC* (uspIC). Throughout this paper, uspIC will refer to the whole localization framework whilst spIC denotes the scan matcher module.

The uspIC is compared to other, previously existing, approaches. The results show the quality of our approach and the superiority of our proposal with respect to the existing underwater scan matching approaches.

## 2. The Mechanically Scanned Imaging Sonar

The scan matching approach presented in this paper has to explicitly deal with the particularities of MSIS sensors. In order to properly handle these particularities it is important to understand the behavior of these sensors. To this end, this section briefly explains the basics behind the acquisition of acoustic images by means of an MSIS. A comprehensive description of these sensors is available in [34]. Although most of the provided description is general to all MSIS, some details are specific to the Tritech Miniking Imaging Sonar, which is the particular MSIS used in our experiments.

### 2.1. Basic Operation

**Figure 1.**
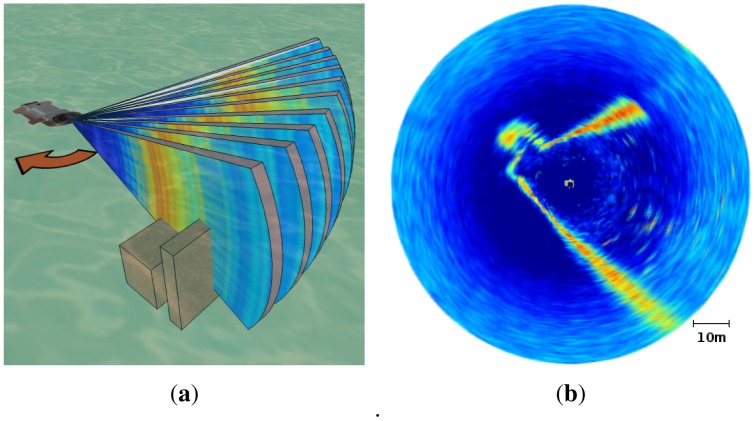
**(a)** Scanning process of an MSIS. The two boxes represent obstacles in the acoustic wave path; (**b**) Example of acoustic image. The colors depict the echo intensities, ranging from blue (low echo intensity) to red (high echo intensity).

An MSIS mechanically rotates the sensing head through a set of predefined angular increments. In our particular case, the sensing head is able to rotate 360° in steps of 1.8°. At each angular position, an ultrasonic fan-shaped beam is emitted. This acoustic beam travels in water and, eventually, collides with one or more objects in the environment, being totally or partially echoed back to the transducer. If the signal is only partially echoed back, which is quite usual, the remaining signal continues traveling and new echoes can be produced. As echoes are received, their amplitude is registered together with the distance at which they were produced, computed by measuring the time of flight. Each of these echo amplitude measurements associated to a range is called a *bin*. The set of all the bins gathered for a specific transducer orientation is referred to as *beam*. In our configuration, each beam is composed of 500 bins and covers a maximum range of 50 m. The set of all the beams gathered during a 360° rotation of the transducer head is referred to as *acoustic image* which, in our case, is composed of 200 beams. Figure 1(a) illustrates the MSIS scanning process. An example of acoustic image is provided in Figure 1(b).

### 2.2. Acoustic Image Interpretation

The basic MSIS operation is similar to other scanning devices, such as laser range finders. However, there are some aspects in the MSIS operation that deserve special attention as they clearly influence the way in which their measurements have to be interpreted. Also, these aspects make this sensor much more problematic to perform localization than a terrestrial laser range finder. Next, these particularities and their effects on the acoustic image interpretation are explained.

In general, gathering an acoustic image takes some time due to the time of travel of the sound that limits the sensing head's rotation speed. Let this time be referred to as *scan time*. For example, using our particular configuration, the scan time is larger than 13 seconds. If the sensor is attached to a mobile robot, the robot motion during the scan time can not be neglected. This induces a distortion in the acoustic image because, as the robot moves, the relative position of the sensor with respect to surrounding obstacles changes while the acoustic image is being built. Thus, contrarily to cameras or laser range finders, the image cannot be considered a snapshot of the environment. Accordingly, some processing has to be performed on the acoustic image to compensate this motion induced distortion.

**Figure 2.**
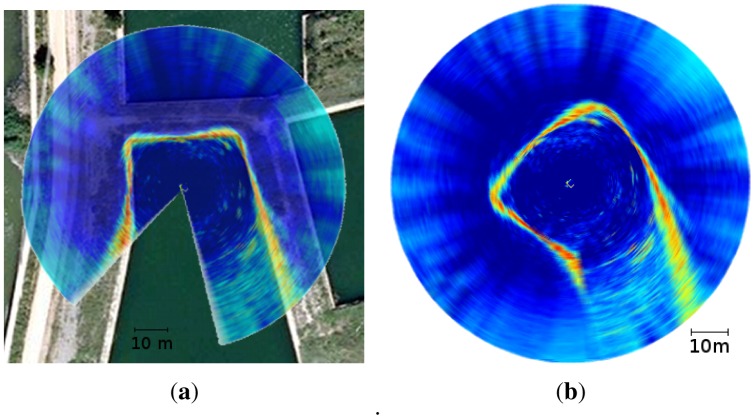
**(a)** A scan as it *should* look like, overlaid to a satellite view of the environment where the data was gathered; (**b**) The scan as it is obtained due to the motion induced distortion.

Figure 2 illustrates this problem. In this example, the robot was rotating while gathering the data in the environment shown in Figure 2(a). The expected acoustic image is shown in the same figure. However, as the robot was rotating, the obtained measurements are those shown in Figure 2(b). Accordingly, some processes are necessary to correct this distortion.

**Figure 3.**
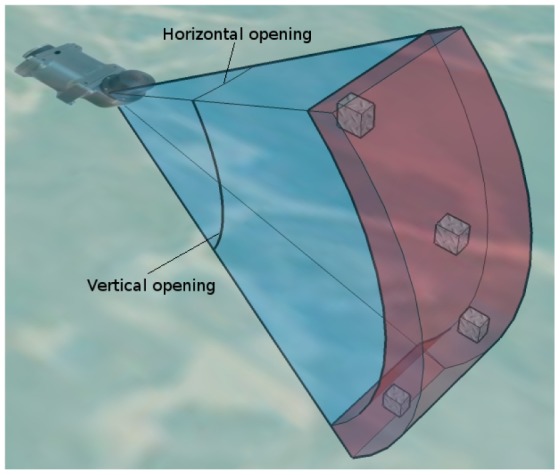
Uncertainty in object detection. All the depicted obstacles (boxes) produce the same measurement (in red) as they are at the same distance to the sensor.

A second aspect that influences the acoustic image interpretation is the shape of the ultrasonic wave. The ultrasonic wave expands with time, and, thus, it can not be considered a thin, pencil like, beam. The resulting shape depends on the specific sensor, and is generally modelled as a wedge defined by the expansion rates over the two axes perpendicular to the wave motion. These expansion rates are commonly represented by the two angles that define the wedge. From now on, let us assume that the sensor mechanical rotation happens in a plane parallel to the floor and let us refer to aforementioned two angles as the horizontal and vertical openings. Figure 3 illustrates this model.

The beam shape is responsible for two effects. First, when an object is detected, its exact location is unknown. The only knowledge we have is that the object lies somewhere in a region of equidistant points inside the wedge. The second effect is that different obstacles located in the aforementioned region of equidistant points will be indistinguishable and the sensor cannot decide if there is one or more obstacles inside that region. Both effects are illustrated in Figure 3. It can be observed how all the boxes produce the same echo, making it impossible to know their exact position (only the range is known) and, thus, being indistinguishable between them.

The effects of the uncertainty in object detection depends on the two openings: the smaller they are, the lower the uncertainty. The Tritech Miniking has a vertical opening of 40° and an horizontal opening of 3°. Thus, the detection accuracy is quite good in the horizontal plane and has a very important uncertainty in the vertical plane. As our goal is to perform 2D localization in the horizontal plane, the vertical opening barely influences the process and the horizontal opening is small enough to obtain reasonably accurate acoustic images.

A final aspect that deserves attention is related to our specific goal, which is scan matching. In general, scan matchers operate on point clouds such as those provided by terrestrial laser range finders. However, the MSIS does not provide a point cloud but an acoustic image. Thus, if scan matching has to be performed, the acoustic image has to be processed in order to obtain a point cloud representing the most significant objects present in the acoustic image.

There are some more aspects, such as multiple reflections, full reflections or shadows that influence the acoustic image formation but are not especially relevant in the context of this study. The reader is directed to [34] for a detailed explanation of such effects.

## 3. Problem Statement and Notation

### 3.1. Scan Matching

Scan matching algorithms require two consecutively gathered sets of range measurements called *scans*. Let *S_ref_* = {*q*_1_,*q*_2_,…,*q_n_*} be a set of *n* measurements gathered at frame *A*, which is called the *reference scan*. Let *S_cur_* = {*p*_1_,*p*_2_,…, *p_m_*} be a set of *m* measurements gathered at frame *B*, which is called the *current scan*. The aim of scan matching is to compute the relative displacement and rotation of the coordinate frame *B* with respect to *A* so that the overlap between *S_ref_* and *S_cur_* is maximized. This relative displacement and rotation constitutes the estimate of the robot motion from *A* to *B* and is denoted by $x_{B}^{A}$.

The measurements in *S_ref_* and *S_cur_* can be represented in different ways, depending on the specific scan matching implementation. For example, the basic ICP and IDC algorithms represent the measurements by their Cartesian coordinates with respect to the corresponding coordinate frame. The proposal of this paper is similar to spIC. The measurements in both scans are represented by normal distributions. The mean vectors represent the Cartesian coordinates of the measurements and the covariances provide information about their uncertainty.

It is common to model the scan matching error as a normal distribution. Therefore, we will represent the scan matching estimate as a multivariate normal distribution $x_{B}^{A}=N\left({\hat{x}}_{B}^{A},P_{B}^{A}\right)$. As $x_{B}^{A}$ is a rototranslation in the plane, the mean vector ${\hat{x}}_{B}^{A}$ has the form [*x,y,θ*]*^T^*, where *x* and *y* represent the translation and *θ* represents the rotation. Accordingly, the covariance $P_{B}^{A}$ is a 3×3 matrix. The obtention of the parameters of the aforementioned normal distribution depend on the specific scan matching approach under consideration. For example, [35] deals with the obtention of this covariance for the IDC algorithm, [36] provides a closed form solution for the ICP covariance and some details regarding the covariance of the spIC are provided by [37].

### 3.2. Scan Matching with an MSIS

**Figure 4.**
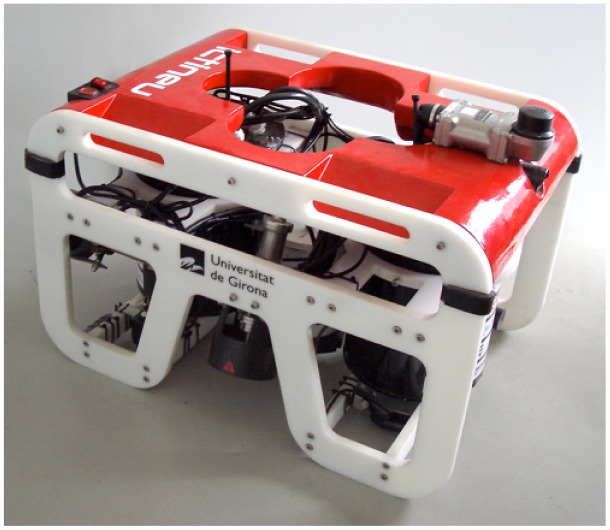
The Ictineu AUV, developed in the University of Girona. (Source: http://cirs.udg.edu/).

The experiments conducted in this paper have been performed using the sensor data gathered by the *Ictineu AUV* (see Figure 4). This AUV was designed and developed in the University of Girona (see [29] for more details). Among other sensors, the Ictineu is endowed with a *SonTek Argonaut Doppler Velocity Log* (DVL), which measures the velocities of the unit with respect to bottom and water at a frequency of 1.5 Hz. A low-cost *Motion Reference Unit* (MRU), the XSens MTi, is also used. This sensor provides absolute attitude data by means of compass and inclinometers at a rate of 10 Hz. The Ictineu is also endowed with the aforementioned MSIS, the *Tritech Miniking Imaging Sonar*.

As stated previously, performing scan matching with an MSIS has two important requirements. First, acoustic images need to be converted to range scans. Second, the effects of the motion induced distortion have to be taken into account. In order to meet these requirements, additional processes are necessary.

Our proposal is as follows. First, each new beam provided by the MSIS is processed to extract the range information. Let this process be named the *beam segmentation*. Also, the information provided by the DVL and the MRU is used to obtain rough pose estimates by means of *dead reckoning*. Both the obtained range information and the dead reckoning estimates are stored in two buffers, called *readings history* and *transformations history* respectively. When a full acoustic image has been gathered, the information in the transformations history is used to compensate the robot motion and build a range scan using the readings in the readings history. Let this process be called the *scan building*. When two consecutive scans have been built in this way, the scan matching is executed. The proposal in this paper is to use the spIC to perform the scan matching, although other scan matching algorithms can be easily used thanks to the uspIC framework. The motion estimate provided by the scan matching is used to correct the transformations history. It is the so-called *pose correction* process. Thanks to this, the robot pose can be continuously computed from the data in the transformations history by means of the *pose extraction* process.

**Figure 5.**
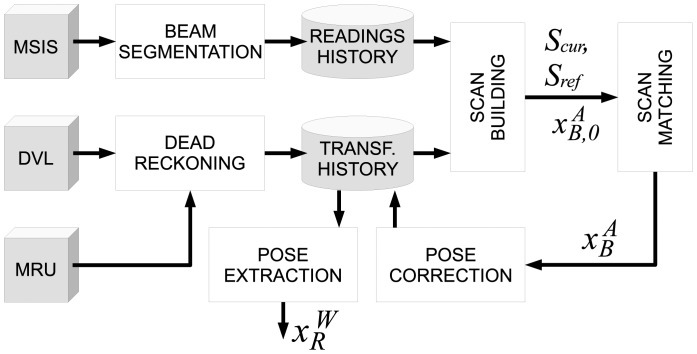
Overview of the uspIC. The notation is explained throughout the paper.

Figure 5 summarizes our proposal. The rest of the paper is devoted to describe the above-mentioned processes except for dead reckoning. Dead reckoning is performed by means of an *Extended Kalman Filter* (EKF) that fuses DVL and MRU data. A detailed description of this EKF is available in [34].

## 4. Beam Segmentation

**Figure 6.**
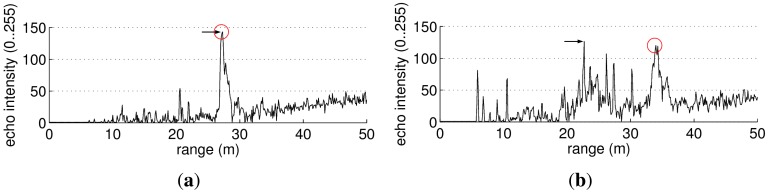
Two different beams. The circle marks the bin where the largest obstacle is located. The arrow points to the largest echo intensity. (**a**) A simple situation; (**b**) A more complex situation.

**Figure 7.**
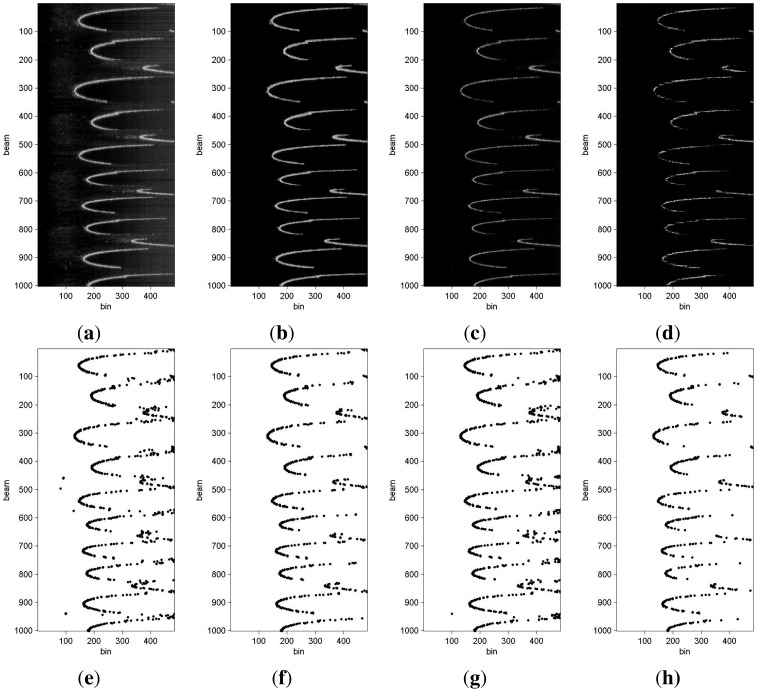
Beam segmentation examples with sets of acoustic images, (**a**) Original image; (**b**) Image processed with method 1; (**c**) Image processed with method 2; (**d**) Image processed with method 3; (**e**) Ranges extracted from original image; (**f**) Ranges extracted from method 1; (**g**) Ranges extracted from method 2; (**h**) Ranges extracted from method 3.

Our goal is to obtain range scans instead of the beams as they are provided by the MSIS. Accordingly, the beam segmentation is in charge of computing the distance from the sensor to one relevant obstacle in the beam. Although in some cases, this distance corresponds to the bin with the largest intensity value (Figure 6(a)), in some other cases it does not (Figure 6(b)), mainly because of noise and spurious measurements. The first column in Figure 7 shows some acoustic images as they are provided by the sensor and the result of selecting the maximum intensity bin for each beam. It can be observed that several spurious ranges are selected.

To deal with these problems, different approaches have been adopted in the literature. For example, [38] performs the beam segmentation using simple thresholding. This approach, as stated previously, is prone to errors and produces false ranges. The study [33] is also based on thresholding to detect peaks on each beam, but improves the results by using a minimum distance between peaks criterion. A more sophisticated approach to extract information from the acoustic images using a voting schema in the Hough space is proposed in [34]. However, this approach is aimed at feature-based SLAM and, thus, it searches high level features such as lines instead of the range measurements which are required for scan matching.

In the context of this study, the following three methods to process MSIS data have been designed, implemented and tested [28].

### 4.1. Method 1: Enhancement and Image Processing Techniques

The goal of this method is to isolate the high intensity areas of the acoustic image produced by obstacles in the environment and, thus, to remove the spurious measurements due to sensor noise or suspended particles in water, among others. In this case, the acoustic image is modified directly applying standard image processing techniques. The acoustic image is represented in an angle *vs.* range (beam *vs.* bin) space and each bin is considered to be a gray scale pixel for the image processing algorithms. Thus, this method operates without taking into consideration any of the acoustic image formation particularities. The process consists of three steps:

1. **Contrast enhancement:** The contrast of the gray scale image is enhanced, so that differences between high and low echo intensities are emphasized.
2. **Thresholding:** The resulting image is converted to a binary image by thresholding.
3. **Morphological operations:** The resulting binary image is modified by consecutively applying two morphological operators: closing and pruning. In this way, small objects in the image, which are likely to correspond to spurious measurements, are removed.

The result of this method is a binary image which is used as a mask over the original acoustic image. Among the remaining bins after applying the mask, the one with the maximum intensity is selected for each beam. The second column in Figure 7 shows some acoustic images processed according to this method and the ranges extracted from this image. The differences between this method and a simple selection of the largest bin for each beam (first column) can be clearly appreciated. Spurious measurements are almost removed when using this method.

### 4.2. Method 2: Range Dependant Enhancement

In general, the acoustic signal attenuates with the traveled distance. To compensate this effect, most sonar sensors, such as the one used in our experiments, use *Time Variable Gain* (TVG) methods to enhance the received signal depending on the time of flight. However, we have observed that, even with the TVG, the acoustic signal attenuation still appears as a loss of contrast depending on the range when the acoustic image is represented as a visual image. This can be appreciated in Figure 7(a), where the image contrast is lower on the right side of the image, which corresponds to larger ranges. The aim of this method is to alleviate the problems due to this range dependent loss of contrast. Our proposal is to enhance each bin individually depending on its range: the larger the range, the more the contrast is enhanced.

When this process has been executed, there are two possible ways to obtain the ranges. On the one hand, the resulting image can be binarized and the binary image can be used as a mask over the original image to select, among the remaining bins, the largest bin value for each beam. The range dependent contrast enhanced image and the resulting ranges are shown in the third row of Figure 7. Results are slightly better than simple maximum selection, but clearly worse than method 1. The second way to obtain ranges using this method is to apply the thresholding and morphological operations of method 1 over the range dependent contrast enhanced image. In this case, results are slightly better than method 1, especially for large ranges, at expense of higher computational cost.

### 4.3. Method 3: Dynamic Thresholding

**Figure 8.**
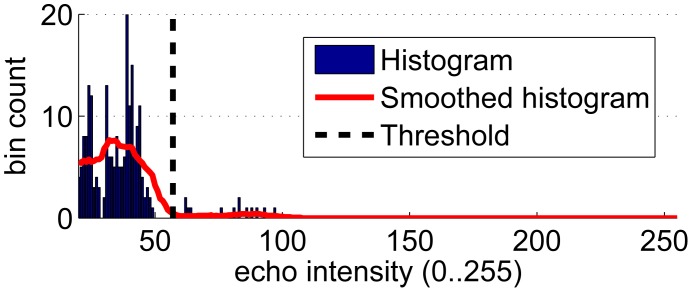
An example of the threshold selection process.

This proposal is as follows. When the MSIS provides a new beam, the beam segmentation process obtains the corresponding range measurement by means of the following three steps:

1. **Thresholding:** An echo intensity threshold is dynamically selected as follows. Firstly, the histogram of echo intensities corresponding to the beam under analysis is computed. Secondly, the histogram is smoothed. Afterwards, the threshold is located at the largest echo intensity value that locally minimizes the smoothed histogram. Figure 8 exemplifies the threshold selection process. In this way, the threshold separates two clearly defined areas in the echo intensity space. Finally, those bins whose intensity is below the threshold are discarded.
2. **Erosion:** The remaining bins are eroded. That means that those bins that, after the thresholding, do not have another bin in their immediate neighborhood are removed. The purpose of this step is to remove spurious measurements.
3. **Selection:** At this point, it is usual that a single cluster of bins remains. The bin with the largest echo intensity value is selected, and the distance corresponding to this bin represents the range value for the beam under analysis. Let the point corresponding to this range be named the *range reading*.

An example of this method applied to some acoustic images is shown on the top of the last column in Figure 7. On the bottom of the same column, the selected ranges are shown. It can be observed that this method removes almost all spurious measurements, similarly to method 1, but also discards valid measurements at large ranges.

However, method 3 provides important benefits when used to perform scan matching. First, it is simple and fast, being very easy to implement and requiring very low computation. Second, it operates on a beam basis, whereas the other two require full acoustic images. Thanks to this, the CPU usage is more homogeneous than in the other methods, which perform their task when the acoustic image has been obtained and do nothing when the data is being gathered. Finally, the experiments suggest that the ratio between the quantity and the quality of the obtained ranges lead to better localization results when used to perform scan matching. Accordingly, the ranges selected with this method are those used to build the scans by means of the scan building process.

## 5. Scan Building

As stated previously, the MSIS data cannot be treated as a synchronous snapshot of the world. Instead, the sonar data is actually acquired whilst the AUV is moving. Thus, the robot motions during the sonar data acquisition have to be taken into account in order to correct the induced distortion. The *scan building* epitomizes this idea. Roughly speaking, scan building is in charge of grouping range readings to conform a scan usable to perform scan matching.

The scan building, and so the scan matching, can be performed in several ways depending on the required temporal resolution and the available computational resources. For example, a sliding window could be used to perform scan matching every time a new beam is acquired. In this way, scan matching estimates would be available at high frequency at the cost of high CPU usage. Another approach is to perform scan building and scan matching only when a fully new acoustic image is obtained. With this approach, the temporal resolution is reduced and, between scan matching estimates, the vehicle has to rely on dead reckoning. However, the CPU usage is drastically reduced. Also, all the described beam segmentation methods can be used in this case, whilst only method 3 is useful in the sliding window case.

Our proposal is to perform scan building, and so scan matching, every time a fully new acoustic image has been gathered. Nevertheless, depending on the desired scan matching frequency and computational resources, this could easily be scaled to have scan matching estimates every, for instance, half acoustic image or even every new beam.

### 5.1. Modeling the Range Readings

The range readings provided by the beam segmentation constitute the range information used to build the scans. Our proposal is to augment this range data with information about its uncertainty, which comes, basically, from two sources: on the one hand, the uncertain knowledge of the robot motion while gathering MSIS data; on the other hand, the uncertain location of the detected objects with respect to the MSIS, as explained in Section 2.2. This augmented data will be modelled by a normal distribution. This section discusses how this model can be constructed from the range information provided by the beam segmentation process, the robot pose and a simple sonar model.

Let $r_{t}=N\left({\hat{r}}_{t},\sigma_{t}^{2}\right)$ denote a measurement obtained at time *t* in form of *random Gaussian variable* (RGV). Let this measurement be represented with respect to a coordinate frame centered on the MSIS and having the *x* axis aligned with the beam acoustic axis at time *t*. In this case, the mean vector has the form *r̂_t_* = [*ρ_t_*, 0]*^T^*, where *ρ_t_* denotes the range reading provided by the beam segmentation at time *t*. The covariance matrix $\sigma_{t}^{2}$ is as follows:

(1) $$\sigma_{t}^{2}=\left[\begin{array}{cc} \sigma_{xx}^{2} & 0\\ 0 & \sigma_{yy}^{2} \end{array}\right]$$

where *σ_xx_* models the range uncertainty and $\sigma_{yy}=\frac{\rho t}{2}\tan\left(\frac{\alpha }{2}\right)$ models the angular uncertainty of *α* degrees. The range uncertainty *σ_xx_* depends on factors such as the sensor resolution, which is 10 cm in our case, and the beam segmentation characteristics, such as the mask sizes used for image operations in beam segmentation methods 1 or 2 or the erosion radius and the specific beam shape, which affects the threshold selection, in method 3. As it is very difficult to quantify these effects, *σ_xx_* has to be obtained experimentally. The angular uncertainty *α* is tightly related to the beam's horizontal opening, and can be obtained from the MSIS specification.

Let *z_t_* represent the measurement *r_t_* with respect to the robot coordinate frame. It is straightforward to obtain *z_t_* from *r_t_* and the MSIS beam angle at time *t*. For the sake of simplicity, henceforth the *z_t_* will be referred to as the sonar readings.

### 5.2. The Scan Building Process

The sonar readings have to be stored in the so-called *readings history* so that they can be easily accessed by the scan building process. The readings history at time *t* contains the most recent *N* sonar readings gathered until time *t*. It is defined as follows:

(2) $$RH_{t}=\left\{z_{t-N+1,\cdots ,}z_{t-2,}z_{t-1,}z_{t}\right\}$$

The value of *N* has to be decided so that *RH_t_* can store two consecutive full 360°scans. In our particular configuration a full MSIS scan is composed of 200 beams. Thus, *N* is set to 400. If beam segmentation methods 1 or 2 are used, sonar readings will be included in *RH_t_* in blocks of 200 every time a full acoustic image has been obtained. To the contrary, when using method 3, sonar readings will be included in the readings history individually after each beam has been gathered.

Let *x̄_t_* denote the robot motion (displacement and rotation) from time step *t* − 1 to time step *t*. This robot motion is modeled as a RGV. Although the MSIS and the dead reckoning sensors (DVL and MRU) do not necessarily operate synchronously, it is trivial to obtain the *x̄_t_* from the dead reckoning EKF.

Similarly to the readings history, let the *transformations history* be defined as a history of the most recent *N* robot motions. That is,

(3) $$TH_{t}=\left\{{\bar{x}}_{t-N+1,\cdots ,}{\bar{x}}_{t-2,}{\bar{x}}_{t-1,}{\bar{x}}_{t}\right\}$$

As the AUV is moving while acquiring the scan, each reading in *RH_t_* may have been obtained at a different robot pose. The goal of the scan building process is to represent each reading in one scan with respect to a common coordinate frame.

Let us denote by *z_i,j_* the measurement *z_i_* ∈ *RH_t_* represented with respect to the robot pose at time *j*, where *t* − *N* + 1 ≤ *i* ≤ *t* and *t* − *N* + 1 ≤ *j* ≤ *t*, being *t* the current time step. *z_i,j_* can be computed as follows:

(4) $$z_{i,j}=\{\begin{array}{ll} z_{i} & i=j\\ {\bar{x}}_{j+1}⊕{\bar{x}}_{j+1}⊕\cdots ⊕{\bar{x}}_{i}⊕z_{i} & j<i\\ \left(⊖{\bar{x}}_{j}\right)⊕\left(⊖{\bar{x}}_{j-1}\right)⊕\cdots ⊕\left(⊖{\bar{x}}_{i+1}\right)⊕z_{i} & i<j \end{array}$$

where the operators ⊕ and ⊖ denote the compounding and inversion operations as described in [39]. Throughout this paper, to ease notation, the operator ⊕ will be used with the following addition with respect to its original definition: if the right-hand operand is a set of points, the operator is applied individually to each point and, thus, it returns the resulting set of points.

The robot motions involved in the Equation (4) are those in *TH_t_*. Hence, by means of this equation, each reading in *RH_t_* can be represented with respect to any coordinate frame referenced in *TH_t_*. Next, it has to be decided which coordinate frame choose to build a scan. The chosen coordinate frame corresponds to the central position of the trajectory followed by the robot when collecting the readings involved in the scan.

The central position has been chosen for two main reasons: on the one hand, because of the similarity to the scans generated by a laser range finder; on the other hand, in order to reduce the maximum uncertainty of each reading with respect to the reference frame. Thus, every time the MSIS performs a 360°scan, *S_cur_* is built as follows:

(5) $$S_{\text{cur}}=\left\{z_{i,t,c},\forall i,t-\frac{N}{2}<i\leq t\right\}$$

where *t_c_* corresponds to the time step at which the robot was at the central position of the trajectory followed while the MSIS acquired the scan.

**Figure 9.**
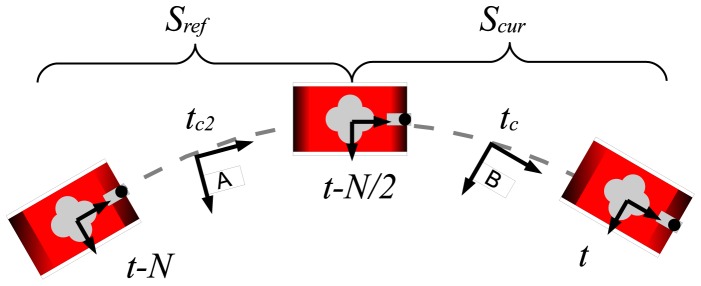
The scan building coordinate frames.

In order to build the reference scan, the measurements that took part in the construction of *S_cur_* in the previous scan matching execution are used. Therefore, the reference scan has the following form:

(6) $$S_{\text{ref}}=\left\{z_{i,tc2},\forall i,t-N<i\leq t-\frac{N}{2}\right\}$$

where *t*_*c*_2__ corresponds to the time step in which the robot was at the central position of the trajectory followed while the MSIS acquired the scan data. Figure 9 graphically depicts the location of the coordinate frames and time steps used during the scan building process.

**Figure 10.**
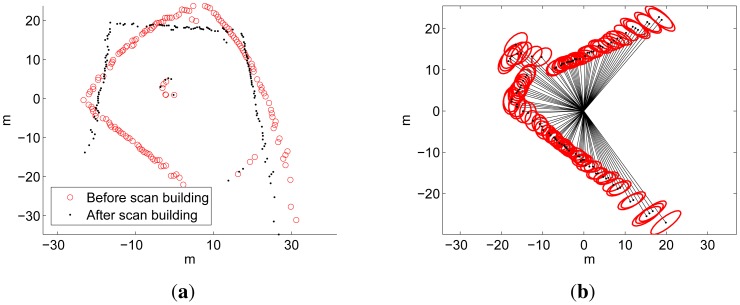
Example of the scan building process. (**a**) A scan before and after the scan building; (**b**) One scan showing the 95% confidence ellipses for each reading.

Figure 10(a) illustrates the result of the scan building by showing a scan before and after the scan building. The readings shown correspond to the acoustic images in Figure 2. Additionally, the sonar measurements, as well as the robot motions, have been modeled by means of normal distributions. This aspect of the scan building is shown in Figure 10(b), where the 95% confidence ellipses of the resulting normal distributions are shown. These normals model the sonar uncertainty, but also take into account the AUV motion uncertainty, which has been included in the scan by Equation (4). This data corresponds to the acoustic image in Figure 1(b).

It is important to emphasize that, due to the pose correction, which is described later in this paper, the robot motions stored in the transformations history may change. As a consequence, *S_ref_* has to be built at each scan matching execution by means of Equation (6). In other words, *S_cur_* is not directly used in the next scan matching execution as *S_ref_* because of possible changes in the transformations history between scan matching executions.

When the scan building has built *S_ref_* and *S_cur_*, the scan matching process is ready to be launched.

## 6. The uspIC

### 6.1. The ICP-Based Approach

The sonar scan matching process is in charge of finding the displacement and rotation between the coordinate frames of the two scans built by the scan building. The sonar scan matching approach adopted in this paper is the *sonar probabilistic Iterative Correspondence* (spIC), which follows the same algorithmic structure of ICP. Thus, it belongs to the ICP-based family of algorithms. Consequently, the ICP-based approach is firstly described.

Generally speaking, the ICP algorithm consists of an iterative process to estimate the robot displacement and rotation that maximize the overlap between two consecutive sensor scans by establishing correspondences involving points in the two scans. Each ICP iteration consists of two steps: the *data association* step and the *minimization* step, which are summarized next.

1. **Data association:** This step is in charge of associating each reading in the reference scan with another one in the current scan. This association is performed according to a proximity criterion: given one reading in the reference scan, the closest one in the current scan is selected. Each association is usually referred to as a *correspondence*. The specific distance used to establish correspondences constitutes the main difference between different ICP-based approaches. The ICP algorithm makes use of the Euclidean distance. As a consequence of this, the ICP considers that the readings can be properly modeled by points. Because of this, the term *point* and the term *reading* will be used interchangeably when referring to the ICP.
2. **Minimization:** The goal of this step is to find the robot motion that maximizes the overlap between the points in the two scans which have been selected during the data association step. This is accomplished by computing the robot motion that minimizes the sum of squared distances between pairs of associated points. Thus, the results of the minimization step are strongly dependent on the data association step because the only readings taken into account are those selected by the data association. If the data association performs some wrong correspondences or omits some useful ones, the minimization step may converge to a wrong estimate.

The ICP iterates these two steps until a certain convergence criterion is met. The proposal by [19] is to iterate until the changes in the least squares error is sufficiently small. However, the specific convergence criterion used to terminate the iterations is not important. As a matter of fact, even the study by Lu and Milios states that, in practice, performing a fixed number of iterations is sufficient.

The main difference between the ICP and the spIC is the distance criterion. The ICP uses the Euclidean distance whereas the spIC utilizes the Mahalanobis distance in order to take into account the statistical information stored in the scans. At the extent of the author's knowledge, there are only three studies, prior to this one, based on the use of such distance in the scan matching context: firstly, the pIC proposed by [21], which is based on the use of a laser range finder; secondly, the spIC, which is designed to be used with terrestrial ultrasonic range finders; finally, the MSISpIC proposed by [33], which constitutes an adaptation of the pIC to be useable with the data provided by an underwater MSIS. Both the pIC and the MSISpIC approximate each set of correspondences by a normal distribution. This approximation, as discussed in [30], is problematic when used in the context of terrestrial ultrasonic range finders. That is why the uspIC scan matcher is based on spIC, and not in the pIC or the MSISpIC. The experimental results will show the benefits of this point of view.

Next, a detailed description of the spIC data association and minimization processes is provided.

### 6.2. Data Association

To ease notation, let the readings in *S_cur_* and *S_ref_* be modeled as normal distributions of the form *p_j_* = *N*(*p̂_j_, P_j_*) and *q_i_* = *N(q̂_i_, Q_i_)* respectively. These normal distributions have been generated during the scan building. Let the $x_{B,k}^{A}$ represent the scan matching estimate at iteration *k*. Also, let $x_{B,k}^{A}$ be modeled by the normal $N\left({\hat{x}}_{B,k}^{A},P_{B,k}^{A}\right)$. The value for the first iteration, $x_{B,0}^{A}$, is obtained from the transformations history as follows:

(7) $$x_{B,0}^{A}={\bar{x}}_{t_{c2}+1}⊕{\bar{x}}_{t_{c2}+2}⊕\ldots ⊕{\bar{x}}_{t_{c}}-1⊕{\bar{x}}_{t_{c}}$$

To decide whether, at iteration *k*, a correspondence can be established or not between *q_i_* ∈ *S_ref_* and *p_j_* ∈ *S_cur_* given the motion estimate $x_{B,k-1}^{A}$, the Mahalanobis distance is used. The squared Mahalanobis distance between *q_i_* and *p_j_* given the motion estimate $x_{B,k-1}^{A}$ is defined as follows:

(8) $$D^{2}\left(x_{B,k-1}^{A},q_{i},p_{j}\right)=h_{i,j}^{T}P_{i,j}^{-1}h_{i,j},$$

where $h_{i,j}\equiv h\left({\hat{x}}_{B,k-1}^{A},{\hat{q}}_{i},{\hat{p}}_{j}\right)$ computes the difference between *q̂_i_* and *p̂_j_*. In order to calculate this difference, *p_j_* has to be previously transformed to the coordinate frame of *S_ref_, A*, by means of $x_{B,k-1}^{A}$. Thus, the function *h, q, p;* is computed as follows:

(9) $$h(x,q,p)=\left[\begin{array}{c} x_{x}+p_{x}\cos x_{\theta }-p_{y}\sin x_{\theta }\\ x_{x}+p_{x}\cos x_{\theta }+p_{y}\sin x_{\theta } \end{array}\right]-\left[\begin{array}{c} q_{x}\\ q_{y} \end{array}\right]$$

where *x* = [*x_x_, x_y_, x_θ_*], *q* = [*q_x_, q_y_*]*^T^* and *p* = [*p_x_, p_y_*]*^T^*.

The covariance matrix of *h_i,j_*, $P_{i,j}\equiv P\left({\hat{x}}_{B,k-1}^{A},{\hat{q}}_{i},{\hat{p}}_{j}\right)$, is obtained linearizing *h* around the displacement estimate ${\hat{x}}_{B,k-1}^{A}$ and the points *q̂_i_* and *p̂_j_* as follows:

(10) $$P_{i,j}=\nabla h_{x}P_{B,k-1}^{A}\nabla h_{x}^{T}+\nabla h_{p}P_{j}\nabla h_{p}^{T}+Q_{i}$$

where

$$\nabla h_{x}\equiv {\frac{\partial h(x,q,p)}{\partial x}|}_{{\hat{x}}_{B,k-1}^{A}{\hat{p}}_{j},{\hat{q}}_{i}}={\left[\begin{array}{ccc} 1 & 0 & -p_{x}\sin x_{\theta }-p_{y}\cos x_{\theta }\\ 0 & 1 & p_{x}\cos x_{\theta }-p_{y}\sin x_{\theta } \end{array}\right]|}_{{\hat{x}}_{B,k-1}^{A}{\hat{p}}_{j},{\hat{q}}_{i}}$$

and

$$\nabla h_{p}\equiv {\frac{\partial h(x,q,p)}{\partial p}|}_{{\hat{x}}_{B,k-1}^{A}{\hat{p}}_{j},{\hat{q}}_{i}}={\left[\begin{array}{cc} \cos x_{\theta } & -\sin x_{\theta }\\ \sin x_{\theta } & \cos x_{\theta } \end{array}\right]|}_{{\hat{x}}_{B,k-1}^{A}{\hat{p}}_{j},{\hat{q}}_{i}}$$

The Mahalanobis distance is, under Gaussian assumption, a chi-squared distribution with *dim*(*h*(*x,q,p*)) degrees of freedom. Hence, points are compatible if and only if $D^{2}\left(q_{i},p_{j}\right)<\chi_{2,\alpha }^{2}$, where *α* is the desired confidence level. For each *q_i_* ∈ *S_ref_*, the set of compatible points in *S_cur_* is searched and the closest one, in the Mahalanobis sense, is selected. As a result of this process, the set *C_k_* of correspondences is built as follows:

(11) $$C_{k}=\left\{\left(i,j\right)|q_{i}\in S_{\text{ref}},p_{j}\in S_{\text{cur}},p_{j}=\text{arg min}\;D^{2}\left(x_{B,k-1}^{A},q_{i},p_{j}\right),D^{2}\left(x_{B,k-1}^{A},q_{i},p_{j}\right)<\chi_{2,\alpha }^{2}\right\}$$

**Figure 11.**
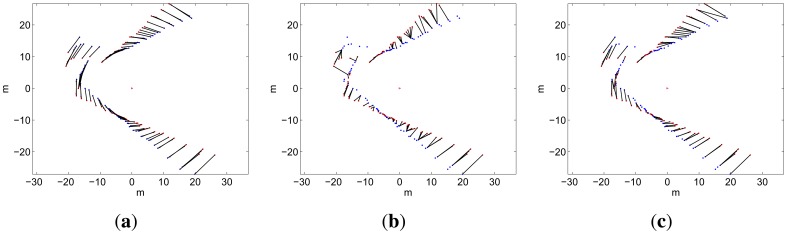
Example of data association. The two scans are misaligned 15 degrees. (**a**) Ground truth correspondences; (**b**) Correspondences according to ICP; (**c**) Correspondences according to spIC.

In order to illustrate the benefits of the spIC approach to data association, Figure 11 is provided. In this example, the same data has been used both for the *S_ref_* and *S_cur_* only to provide a ground truth image. The scans are misaligned 15°. The lines in Figure 11(a) represent the ground truth correspondences. Finally, the correspondences according to ICP and spIC are shown in Figure 11(b,c) respectively. By comparing them with the ground truth, it is clear that the set of correspondences produced by spIC is significantly better than the one produced by ICP. Also, it is clear that spIC is able to capture the rotational error much better than ICP. The superior quality of spIC is mainly due to the fact that it takes into account the sonar and the motion uncertainties.

### 6.3. Minimization

The second step in the scan matching process is the minimization. Its goal is to find the relative displacement $x_{B,k}^{A}$ between the two scans that minimizes the error between pairs of corresponding points. Given *C_k_*, the *spIC error* for a given motion estimate *x* is defined as follows:

(12) $$e_{k}(x)=\sum_{(i,j)\in C_{k}}D^{2}\left(x,q_{i},p_{j}\right)$$

The robot motion ${\hat{x}}_{B,k}^{A}$ searched by the minimization step is the one that makes the spIC error minimum. That is,

(13) $${\hat{x}}_{B,k}^{A}=\arg\min e_{k}(x)$$

Next, a closed form solution for Equation (13) is derived. By linearizing the function *h*, which is involved in the Mahalanobis distance, around the previous scan matching estimate ${\hat{x}}_{B,k-1}^{A}$ and using the first order Taylor approximation, a point *q_i_* ∈ *S_ref_* can be expressed by $\nabla h_{x}{\hat{x}}_{B,k-1}^{A}-h_{i,j}$where ∇*h_x_* denotes the Jacobian in Equation (11). Thanks to this, the spIC error can be rewritten as follows:

(14) $$e_{k}(x)={\left(Jx-A\right)}^{T}Q^{-1}(Jx-A)$$

where *J* and *A* are column vectors of *|C_k_|* elements whose components are such as the form of ∇*h_x_* and $\nabla h_{x}{\hat{x}}_{B,k-1}^{A}-h_{i,j}$ respectively, and *Q* is a block diagonal matrix containing the *P_i,j_*, ∀(*i,j*) ∈ *C_k_*.

By using the orthogonality principle, the solution to Equation (13) is as follows:

(15) $${\hat{x}}_{B,k}^{A}={\left(J^{T}Q^{-1}J\right)}^{-1}J^{T}Q^{-1}A$$

This equation constitutes the closed form solution to compute the mean of $x_{B,k}^{A}$. Consequently, the obtained ${\hat{x}}_{B,k}^{A}$ is ready to be used in further spIC iterations. The obtention of the covariance $P_{B,k}^{A}$ is not a trivial issue and is out of the scope of this paper. Some approaches exist in the literature to deal with this problem. The reader is encouraged to see [35–37] for more information on this subject.

## 7. Pose Correction and Pose Extraction

### 7.1. Pose Correction

The scan matching provides the estimate $x_{B}^{A}$ of the displacement and rotation between the two scan coordinate frames, *A* and *B*. However, the scans have not been obtained at these frames. Instead, the scan readings have been obtained throughout a robot trajectory and the use of *A* and *B* is only a convenient way to represent the MSIS data. The goal of the *pose correction* is to correct the aforementioned robot trajectory so that it fits to the scan matching estimate.

The information about the trajectory followed by the robot during the scan building is available in the transformations history. Accordingly, the goal of the pose correction is to properly include the scan matching estimate into the transformations history. To this end, it has to be taken into account that the composition of the transformations history items from *x̄_tc_*_2+1_ to *x̄_tc_* should be equal to the scan matching estimate. As a matter of fact, the composition of the aforementioned transformations history items was the initial scan matching estimate, as shown in Equation (7).

**Figure 12.**
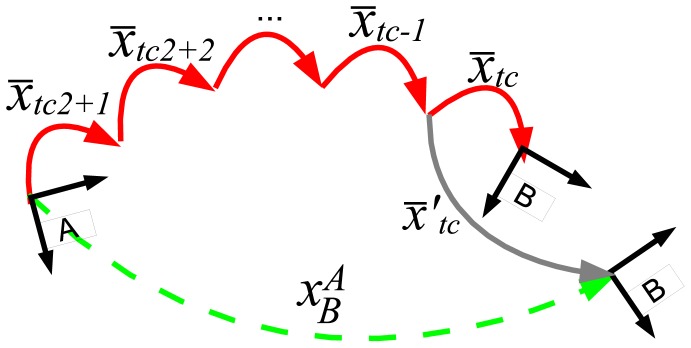
The pose correction process. Only one item in the transformations history is changed to meet the scan matching estimate.

A possible way to do this is to correct each motion estimate in the transformations history according to its uncertainty. This solution is the so-called *trajectory correction* and is described by [30]. The pose correction discussed in this paper is a simplified version of the trajectory correction that runs much faster at the cost of producing slightly less smooth trajectories. Instead of distributing the error correction through all the mentioned transformations history items, the pose correction performs one single change in the transformations history. Figure 12 illustrates this idea.

From this Figure, it is easy to see that the mean of *x̄_tc_* should be corrected to the mean of ${\bar{x}}_{tc}^{\prime }$ as follows:

(16) $${\bar{x}}_{tc}^{\prime }=⊖\left({\bar{x}}_{tc2+1}⊕\cdots ⊕{\bar{x}}_{tc-1}\right)⊕x_{B}^{A}$$

The covariance of the corrected transformations history item, $P_{tc}^{\prime }$, is also needed. However, the covariance of ${\bar{x}}_{tc}^{\prime }$ is not a good approximation because it accumulates the uncertainties of ⊖(*x̄_tc_*_2_*_+_*_1_ ⊕ … ⊕ *x̄_tc_*_−1_) and $x_{B}^{A}$. A possible way to overcome this problem is as follows. To ease notation, let *x_th_* = *N*(*x̄_th_, P_th_*) be defined as *x̄_tc_*_2+1_ ⊕ … ⊕ *x̄_tc_*_−1_. Thus, it is easy to see that:

(17) $$x_{B}^{A}=x_{th}⊕{\bar{x}}_{tc}^{\prime }$$

From this equation, the covariance of $x_{B}^{A}$ can be expressed as follows:

(18) $$P_{B}^{A}=J_{1⊕}P_{th}J_{1⊕}^{T}+J_{2⊕}P_{tc}^{\prime }J_{2⊕}^{\prime }$$

where the terms J_1⊕_ and J_2⊕_ are the Jacobians matrices of the composition, as defined by [40]. Consequently, the covariance matrix $P_{tc}^{\prime }$ can be computed as follows:

(19) $$P_{tc}^{\prime }=J_{2⊕}^{-1}\left(P_{B}^{A}-J_{1⊕}P_{th}J_{1⊕}^{T}\right){\left(J_{2⊕}^{T}\right)}^{-1}$$

This equation can only be used if the eigenvalues of $P_{B}^{A}$ are smaller than those of *P_th_*. Otherwise, the resulting $P_{tc}^{\prime }$ will not be positive definite and, thus, not actually be a covariance matrix. As a matter of fact, in these cases the covariance $P_{tc}^{\prime }$ does not even exist. A possible way to deal with these situations is to leave the covariance unchanged in the transformations history. The experimental results suggest that the error introduced by this simplification is negligible.

Besides, if necessary, the pose correction process can be substituted by the aforementioned trajectory correction.

### 7.2. Pose Extraction

The goal of the pose extraction is to obtain an estimate of the robot pose. As the scan matching estimate has been used to correct the transformations history, the corrected robot pose can be easily obtained, precisely, from the transformations history.

The transformations history contains the last *N* robot motions obtained from dead reckoning and, eventually, corrected by means of the scan matching estimate. When a new motion estimate is obtained from dead reckoning, it is included into the transformations history and, at the same time, the oldest item is discarded. Consequently, the relative position of the first item in the transformations history with respect to a fixed reference frame can be iteratively computed by

(20) $$x_{t}^{W}=x_{t-1}^{W}⊕{\bar{x}}_{t-N}$$

where *x̄_t_*_−_*_N_* represents the item in the transformations history that has been discarded at time step *t*. The term $x_{t}^{W}=N\left({\hat{x}}_{t}^{W},P_{t}^{W}\right)$ denotes the relative position, at time step *t*, of the first item in the transformations history with respect to an earth-fixed coordinate frame *W*. Initially, both ${\hat{x}}_{t}^{W}$ and $P_{t}^{W}$ can be set to zero, meaning that the initial robot pose is the origin of coordinates and that, initially, the robot location is perfectly known.

**Figure 13.**
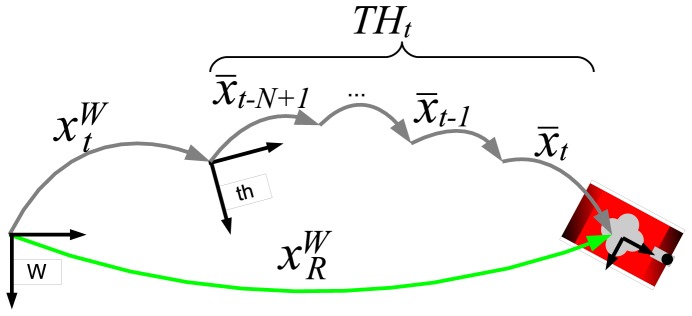
The pose extraction process. The term $x_{R}^{W}$ denotes the robot pose.

The robot pose with respect to the global frame *W* at time step *t* is denoted by $x_{R}^{W}$ and can be computed as follows:

(21) $$x_{R}^{W}=x_{t}^{W}⊕{\bar{x}}_{t-N+1}⊕\cdots ⊕{\bar{x}}_{t-2}⊕{\bar{x}}_{t-1}⊕{\bar{x}}_{t}$$

where the terms *x̄_t_*_−_*_N_*_+1_ to *x̄_t_* are those in the transformations history. As the scan matching estimates have been used to correct the transformations history, computing the robot pose by means of Equation (21) implicitly takes into account the scan matching corrections. The pose extraction is summarized in Figure 13.

## 8. Experimental Results

### 8.1. Experimental Setup

The experimental data used to validate the uspIC was obtained by [29] in an abandoned marina situated near St. Pere Pescador in the Costa Brava (Spain). The Ictineu AUV was tele-operated along a 600 m trajectory at an average speed of 0.2 m/s. The trajectory includes a small loop as well as a 200 m long straight path. The gathered data included measurements from the DVL, the MRU and the MSIS. The DVL was configured to use the bottom velocities. The MRU has been used only to provide compass data. The particular MSIS configuration has been discussed in Section 2. Additionally, a buoy with a GPS was attached to the robot in order to obtain the ground truth. Some more details regarding the experimental setup are provided in [29]. Also, some other authors [33,38,41] have used the same dataset to test their localization and SLAM algorithms.

**Figure 14.**
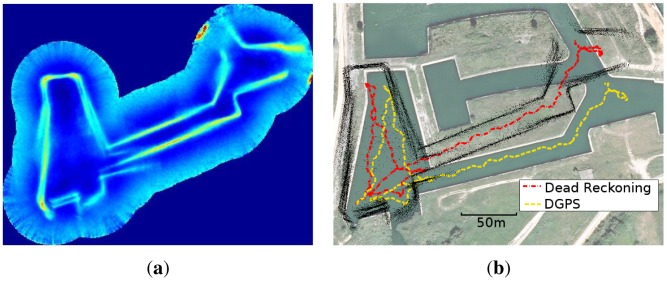
**(a)** The acoustic images drawn according to the dead reckoning; (**b**) Dead reckoning and GPS data overlaid to a satellite view of the environment.

Figure 14(a) shows all the gathered acoustic images combined according to the dead reckoning. The problems of dead reckoning can be appreciated. For example, the data in the entrance to the canal is misaligned due to the suffered drift.

Figure 14(b) shows the dead reckoning and GPS trajectories layered over a satellite view of the area. The problems of drift are clearly visible here, as the dead reckoning trajectory clearly goes outside the water. The black dots represent the MSIS readings after the beam segmentation plotted according to dead reckoning.

In order to evaluate the proposal in this paper, the uspIC is compared to two approaches to scan matching using an MSIS, *i.e.*, MSISpIC [33] and *Underwater Sonar ICP* (usICP). MSISpIC is based on the so-called *scan grabbing*, which is in charge of segmenting the MSIS beams and collecting them to build scans, and the pIC algorithm by [21] to perform the matching. In contrast, usICP consists in performing the same beam segmentation, scan building, pose correction and pose extraction processes described in this paper but changing the scan matching. Instead of the spIC, the usICP makes use of the standard and well known ICP algorithm.

### 8.2. Quantitative Evaluation

**Figure 15.**
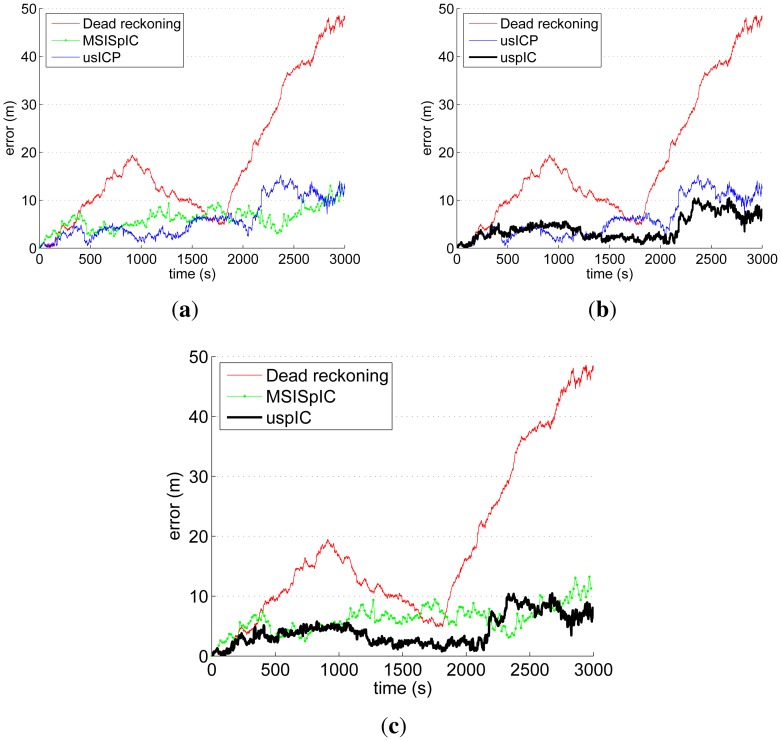
Quantitative evaluation, comparing dead reckoning with (**a**) MSISpIC and usICP; (**b**) usICP and uspIC; and (**c**) MSISpIC and uspIC.

The trajectories estimated by dead reckoning, usICP, MSISpIC and uspIC have been compared to the ground truth provided by the GPS. The comparison consists of measuring, at each time step, the distance from the GPS ground truth to the estimated robot pose according to each of the mentioned methods. The results are shown in Figure 15. Also, Table 1 shows the percentage of the mission time in which each method provided better results than each of the other ones.

Figure 15(a) shows the results for those methods to which the proposal in this paper is compared. It is clear that both the MSISpIC and the usICP are able to provide pose estimates much better than dead reckoning. According to Table 1, the usICP provides better estimates than MSISpIC in 65.28% of the time. Figure 15(b) compares usICP and uspIC. In this case, the uspIC error is below the usICP one in 69.53% of the time. Also, the uspIC improves dead reckoning in the 97.31% of the time. The remaining 2.69% in which uspIC results are below dead reckoning only happens at the very beginning, when dead reckoning error is still very low. Finally, Figure 15(c) compares the uspIC and the MSISpIC and shows that the uspIC provides better pose estimates than MSISpIC in the 73.62% of the time. According to this, the scan matching approach that provides better results during the most part of the time is the uspIC, followed by the usICP and the MSISpIC, in this order.

Table 2 shows the mean and the maximum improvement of each method with respect to each other. In this table, improvement of method A with respect to method B is measured as the error difference in those cases in which A is better than B. As the table shows all the combinations, the situations where each method performs worse than the others is also shown. For example, it can be observed that in the 2.69% of the time where uspIC provides higher error than simple dead reckoning (Table 1), the maximum improvement of dead reckoning with respect to uspIC was 0.8 m and the mean was 0.3 m. To the contrary, in the 97.31% of the time when uspIC was better than dead reckoning, the maximum improvement reached 44.05 m and the mean was 14.52 m. Thus, Table 2 provides a numerical measure of how better was each method during the percentage of time shown in Table 1.

The mean error, the maximum error and the standard deviation of the absolute error for all the discussed scan matching methods are provided in Table 3. The minimum error is not provided because it is zero for all the methods just at the beginning of the experiment. The uspIC is the algorithm that has the lowest average and maximum error. Regarding the usICP and the MSISpIC, they behave similarly in terms of mean and maximum error.

The standard deviation of the error somehow represents the stability of the localization method. High standard deviations represent frequent changes in the pose quality. From this point of view, the uspIC and the MSISpIC have similar stability, and both are significantly more stable than the usICP. This is consistent with Figure 15, where it can be observed that the usICP error is more irregular than the other ones.

**Figure 16.**
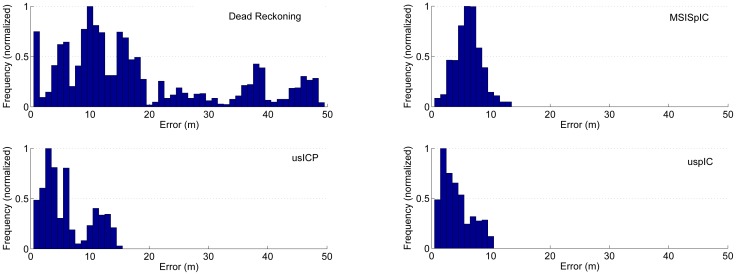
Histogram of the error for dead reckoning, MSISpIC, usICP and uspIC. The error frequency has been normalized for clarity purposes.

Figure 16 shows the histograms of the localization error. The histograms clearly reflect the aforementioned maximum error. However, the histogram also provide information about how this error is distributed. For example, it is clear that the dead reckoning error spreads over a large region but the most part of it is concentrated between 0 m and 20 m of error. It is remarkable that the most part of the usICP error is around the 3 m, which is significantly below the 6 m of the MSISpIC. However, the usICP errors spread over a larger area than MSISpIC, and that is why both approaches have a similar average error. Finally, it is also remarkable that not only is uspIC the method with the lowest maximum error, but also that the most part of the error is concentrated around 2 m.

In our proposal, scan matching is performed every time a fully new acoustic image is available. While each acoustic image is being gathered, the robot has to rely on dead reckoning. As in our particular configuration gathering an acoustic image takes 13 s, the dead reckoning error may increase significantly and, thus, the scan matching correction may be significantly large. In order to show how large are the scan matching corrections, we have measured the differences in the pose estimate from each acoustic image to the next according to dead reckoning and according to uspIC. We have observed that, in mean, each uspIC execution involves a change in the pose estimate of 0.746 m with respect to the dead reckoning one, with a standard deviation of 0.486 m. The maximum correction is 2.636 m.

These changes in the pose estimate when scan matching is executed may be problematic for local navigation. However, they are not for large scale mapping or planning. Thus, for local navigation where the absolute robot pose is usually not relevant, dead reckoning could be used and combined with the scan matching estimates only when absolute pose information is required.

The time consumption of uspIC has also been measured in order to analyze the feasibility of an on-line implementation. The uspIC has been executed on a laptop endowed with an Intel Core Duo at 2.4 GHz running Ubuntu 10.04LTS. The implementation consists of Matlab 7.9 R2009b code except for the uspIC data association and the beam segmentation histogram management which have been coded in C and interfaced with Matlab through MEX functions. We would like to emphasize that Matlab barely takes advantage of the dual core and, thus, the programs are basically executed by one of the two CPU cores.

Table 4 shows the time spent in each of the uspIC tasks during all the mission execution. As the experiment consisted of moving the underwater robot in the marina environment during 53 minutes, the percentages shown in the table denote the percentage of the whole mission actually spent in uspIC computations. It can be observed that the spIC scan matching time only represents a 3.69% of the time, which is lower than the time spent by the dead reckoning EKF. Of course, it has to be taken into account that dead reckoning is performed continuously while spIC is only executed when a fully new acoustic image is available.

It can also be observed that the pose correction and scan building times are almost negligible, whilst the most part of the time consumption is due to the beam segmentation.

One of the most important aspects is that the whole localization process consumes, in mean, the 40.667% of the mission time. Thus, in mean, the CPU is free to perform other tasks during the most part of the time even using a Matlab implementation. Moreover, during the mission execution 218 scans were gathered, meaning that the whole localization process consumes 5.932 s per scan in mean. As this time is significantly lower than the 13 s required to gather a fully acoustic image, it is reasonable to assume that even the Matlab implementation could be used for on-line execution.

### 8.3. Qualitative Evaluation

**Figure 17.**
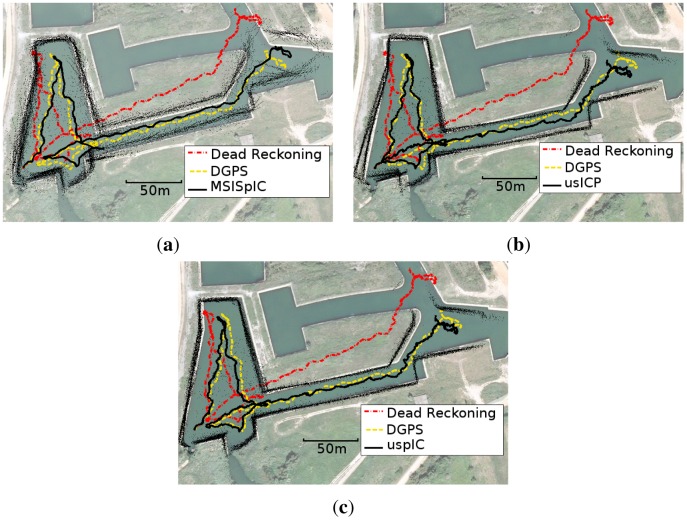
Trajectories corresponding to GPS, dead reckoning and (**a**) MSISpIC; (**b**) usICP; and (**c**) uspIC.

Figure 17 shows the trajectories obtained by MSISpIC, usICP and uspIC and visually compares them to the ones provided by dead reckoning and the GPS ground truth. Moreover, the data is superimposed to a satellite view of the area to provide a clear idea of the quality of the obtained trajectories.

The first thing to be noticed is that the three methods produce a trajectory close to the ground truth, especially when it is compared to the dead reckoning data. Besides, there are some differences that deserve special attention.

Firstly, the MSISpIC (Figure 17(a) has not been able to solve the double wall effect on the left side of the image, although the effect has been significantly reduced with respect to dead reckoning (see Figure 14(b). This effect appears when the AUV returns to a previously visited area with a significant pose error. Also, the data corresponding to the entrance to the canal is misaligned, similarly to dead reckoning, where two entrances to the canal seem to exist.

Secondly, the usICP (Figure 17(b) has, similarly to dead reckoning and MSISpIC, the problem of perceiving a double wall on the left side of the image. However, contrarily to MSISpIC, the usICP has been able to align the data corresponding to the canal entrance. Moreover, it can be observed that the trajectory provided by usICP is slightly shorter than the one provided by MSISpIC. This difference is due to the canal shape. As the canal is almost straight, it is very difficult for an scan matching algorithm to determine the robot motion along the corridor direction.

Moreover, the uspIC (Figure 17(c) is the algorithm that clearly exhibits better results. First, the double wall effect does not appear. Moreover, the canal entrance data is perfectly aligned. Also, at the end of the experiment, the uspIC is the approach whose pose estimate is the closest to the ground truth. It can also be observed that the uspIC data fits the satellite image better than the other methods, especially on the water tank on the left.

**Figure 18.**
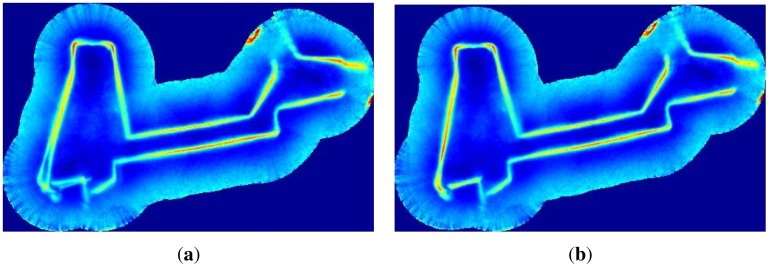
All the gathered acoustic images drawn according to (**a**) usICP; and (**b**) uspIC.

Finally, for the sake of completeness, Figure 18 shows the data provided by the MSIS depicted according to usICP and uspIC trajectories. The same data drawn using the dead reckoning trajectory has been shown in Figure 14.

## 9. Conclusions

This paper presents a novel approach to localize an underwater mobile robot. The localization process, which also relies on some proprioceptive sensors to perform dead reckoning, is focused on the use of an MSIS. When used to perform scan matching, these sensors present two important problems. Firstly, they provide echo intensity profiles instead of the sets of range measurements required in the scan matching context. Secondly, the scan time is not negligible and, thus, the robot motion during the data acquisition has to be explicitly taken into account.

The approach presented in this paper is the so called uspIC. The uspIC deals with the aforementioned problems. On the one hand, the uspIC extracts range information from the echo intensity profiles by means of a beam segmentation process. On the other hand, it groups the extracted range measurements taking into account the robot motion in order to build range scans. Moreover, by adopting a probabilistic scan modeling and matching, it includes statistical information in the scan matching process.

The experimental results compare the uspIC to other previously existing methods. Our approach is compared to the well known and widely used ICP algorithm. It is also compared to the MSISpIC, which is an underwater scan matching method that utilizes the pIC concepts.

The obtained results show an important improvement in the pose estimate with respect to the other tested methods. Some graphical representations of the obtained trajectories have also been provided for the reader to visually compare them.

## Figures and Tables

**Table 1.** Percentage of time where method A provided better results than method B. DR means dead reckoning.

*A/B*	**DR**	**usICP**	**MSISpIC**	**uspIC**
DR	0%	7.94%	16.41%	2.69%
usICP	92.06%	0%	65.28%	30.03%
MSISpIC	83.59%	34.72%	0%	26.38%
uspIC	97.31%	69.53%	73.62%	0

**Table 2.** Mean/maximum improvement, in meters, of method A with respect to method B. DR means dead reckoning.

*A/B*	**DR**	**usICP**	**MSISpIC**	**uspIC**
DR	NA	0.48 m/1.7 m	1.85 m/4.56 m	0.3 m/0.8 m
usICP	13.43 m/40.42 m	NA	2.26 m/6.67 m	1.16 m/4.04 m
MSISpIC	14.88 m/38.86 m	3.7 m/12.03 m	NA	1.65 m/7.13 m
uspIC	14.52 m/44.05 m	3.08 m/5.95 m	3.28 m/8.26 m	NA

**Table 3.** Maximum, average and standard deviation of the error for the tested methods.

**Method/Error**	**Mean**	**Maximum**	**Std. Dev.**
Dead Reckoning	18.32 m	49.03 m	13.64
MSISpIC	6.19 m	13.25 m	2.26
usICP	6 m	15.29 m	4
uspIC	4.21 m	10.47 m	2.5

**Table 4.** Time consumption for each task involved in uspIC. The percentages denote the fraction of time of the whole mission execution corresponding to each task.

**Task**	**Time**	**Percentage**
Dead reckoning	176.031 *s*	5.54%
Beam segmentation	993.139 *s*	31.23%
Scan building	6.751 *s*	0.21%
spIC	117.282 *s*	3.69%
Pose correction	0.0115 *s*	0.0004%
Total	1,293.214	40.667%
